# The age-specific burden and household and school-based predictors of child and adolescent tuberculosis infection in rural Uganda

**DOI:** 10.1371/journal.pone.0228102

**Published:** 2020-01-29

**Authors:** Carina Marquez, Mucunguzi Atukunda, Laura B. Balzer, Gabriel Chamie, Joel Kironde, Emmanuel Ssemmondo, Theodore D. Ruel, Florence Mwangwa, Khai Hoan Tram, Tamara D. Clark, Dalsone Kwarisiima, Maya Petersen, Moses R. Kamya, Edwin D. Charlebois, Diane V. Havlir

**Affiliations:** 1 University of California, San Francisco, Division of HIV, Infectious Diseases and Global Medicine, San Francisco, California, United States of America; 2 Infectious Diseases Research Collaboration, Kampala, Uganda; 3 University of Massachusetts, Amherst, Department of Biostatistics and Epidemiology, Amherst, Massachusetts, United States of America; 4 University of California, San Francisco, Department of Pediatrics, San Francisco, California, United States of America; 5 Stanford University School of Medicine, Palo Alto, California, United States of America; 6 Makerere University College of Health Sciences, School of Medicine, Kampala, Uganda; 7 University of California, Berkeley School of Public Health, Berkeley, California, United States of America; 8 Center for AIDS Prevention (CAPS), University of California, San Francisco, San Francisco, California United States of America; Yeshiva University Albert Einstein College of Medicine, UNITED STATES

## Abstract

**Background:**

The age-specific epidemiology of child and adolescent tuberculosis (TB) is poorly understood, especially in rural areas of East Africa. We sought to characterize the age-specific prevalence and predictors of TB infection among children and adolescents living in rural Uganda, and to explore the contribution of household TB exposure on TB infection.

**Methods:**

From 2015–2016 we placed and read 3,121 tuberculin skin tests (TST) in children (5–11 years old) and adolescents (12–19 years old) participating in a nested household survey in 9 rural Eastern Ugandan communities. TB infection was defined as a positive TST (induration ≥10mm or ≥5mm if living with HIV). Age-specific prevalence was estimated using inverse probability weighting to adjust for incomplete measurement. Generalized estimating equations were used to assess the association between TB infection and multi-level predictors.

**Results:**

The adjusted prevalence of TB infection was 8.5% (95%CI: 6.9–10.4) in children and 16.7% (95% CI:14.0–19.7) in adolescents. Nine percent of children and adolescents with a prevalent TB infection had a household TB contact. Among children, having a household TB contact was strongly associated with TB infection (aOR 5.5, 95% CI: 1.7–16.9), but the strength of this association declined among adolescents and did not meet significance (aOR 2.3, 95% CI: 0.8–7.0). The population attributable faction of TB infection due to a household TB contact was 8% for children and 4% among adolescents. Mobile children and adolescents who travel outside of their community for school had a 1.7 (95% CI 1.0–2.9) fold higher odds of TB infection than those who attended school in the community.

**Conclusion:**

Children and adolescents in this area of rural eastern Uganda suffer a significant burden of TB. The majority of TB infections are not explained by a known household TB contact. Our findings underscore the need for community-based TB prevention interventions, especially among mobile youth.

## Introduction

Tuberculosis (TB) in children and adolescents is an overlooked epidemic with a staggering disease burden.[1] An estimated one million children ages 0–15 years of age fell sick from TB in 2017[2] and 727,000 adolescents ages 12–19 years of age fell sick from TB in 2012.[3] Active TB in youth represents just the surface of a large reservoir of latent TB infections, and an estimated 7.5 million children are infected with TB annually in 22 high TB burden countries.[4] Despite this large TB burden in youth, there remain significant gaps in our understanding of the epidemiology of TB. Age-specific data on the burden and drivers of TB acquisition among both children and adolescents are sparse but needed to guide TB control efforts, especially in rural areas of East Africa.

To date most studies on predictors of child and adolescent TB focus on household exposures and individual-level risk factors, while the role of exposures outside the home remain largely unexplored. Though household exposure is a strong risk factor for child TB, home exposure accounts for less than 20% of new TB cases at the population-level.[5] In South Africa, the relative contribution of community to household exposures shifts as children age into young adulthood[6]. School-age children and adolescents spend the majority of daytime indoor hours in school and other aggregate community-based settings,[7] and characterizing the age-specific risk factors outside of the home is needed in high TB burden areas outside of South Africa.

To address these knowledge gaps, in a community-based sample of children and adolescents ages 5 through 19 years of age (defined in the manuscript as “youth”) living in rural Uganda we sought to: (1) characterize the burden of TB infection in youth by calculating the age-specific prevalence of TB infection, (2) assess individual-level, household-level, and community-level predictors of TB infection among children and adolescents, and (3) to explore age-related variations in the relationship of household TB exposure and prevalent TB infection.

## Methods

### Study design

In this cross-sectional study, from October 2015-December 2016 we performed tuberculin skin testing (TST) on children and adults from 9 communities in Eastern Uganda, living in households participating in a nested household survey in the SEARCH Trial (NCT00948896). The SEARCH trial is a community-based, cluster-randomized “test and treat” trial in 32 rural communities in Uganda and Kenya.[8] The reported incidence rate of TB disease in the communities in Eastern Uganda participating in SEARCH was 58.5 per 100,000 person-years in 2014.[8] For the nested study we randomly sampled from each community 100 households with an adult (≥15 years of age) living with HIV and 100 households that did not have an adult with HIV. Among selected households, TSTs was performed in all household members aged 5 and older.

### Study measures and definitions

Households participating in the nested survey were visited by research assistants up to three times for TST placement. TSTs were performed on the volar aspect of each participant’s forearms, using one dose of tuberculin (Tubersol®). The largest transverse diameter of induration was measured 48–72 hours after placement using the “ball-point” technique. All research assistants underwent a comprehensive training program that included standardized Center for Disease Control training materials [9], and three research assistants placed and read all TSTs in this study. Homes were visited up to three times for TST placement. We defined a positive TST as ≥10mm and ≥5 mm for those with HIV. Receipt of BCG vaccination was defined as the presence of a BCG scar on the deltoid region of the arm or proof of BCG vaccination in the vaccination record.

Participants or their parents, if age <15 years, also completed a brief questionnaire on their TB contact history, history of TB disease, and the WHO tuberculosis symptom screen. History of TB disease was defined by self-report or if the person was listed in the community’s TB registry from 2014–2016. Household TB contact was defined as self-report of ever being exposed to TB in the home or household member with TB disease recorded in the TB registry from 2014–2016. Demographics data, HIV status, school attendance, mobility, and alcohol use were assessed through the larger SEARCH study. We calculated a household wealth index across each community using principal components analysis, based on ownership of livestock (cows, goats, and poultry) and household items (clock, radio, television, phone, refrigerator, bicycle, motorcycle, and electricity).[10] Mobile participants were defined as living outside of the community for six months or more. Having an HIV-infected mother was used as a proxy for perinatal HIV-exposure in HIV-uninfected child. In this manuscript, we refer to participants 5–11 years of age as children, 12–19 year of age as adolescents, and 5–19 years of age as youth.

### Statistical analyses

*TB Infection Prevalence Estimates*: To estimate the population-level age-stratified prevalence of TB infection (defined as a positive TST) we used inverse probability of sampling weighting [11,12] to adjust for (1) oversampling of households with an HIV positive member and (2) incomplete placement of TSTs in sampled households. The probability of household selection was estimated using the community-specific empirical proportions, and the probability of an individual’s participation (within a selected household) was estimated with logistic regression controlling for age, sex, and HIV status. Statistical inference was based on the non-parametric bootstrap, treating the household as the independent unit.

*Predictors of TB Infection*: Our primary outcome is prevalent TB infection, which we defined as a positive TST. Predictor variables were grouped into three categories: individual-level (e.g. age, sex, HIV status), household-level (e.g. household TB contact), and community/school based (e.g. boarding school). To assess the association between potential exposure variables and prevalent TB infection, we calculated unadjusted and adjusted odds ratios with generalized estimating equations (GEE), which accounted for clustering by household, and used inverse weighting to account for incomplete measurements. Adjusted models controlled for age, gender, HIV status, wealth, and household contact with TB disease, and only included other predictors whose univariate association with the outcome yielded a p-value <0.1. All analyses were conducted pooling over and stratified by age-group (5–11 years and 12–19 years). We performed a test of homogeneity to assess for differences across age-group, and considered a p-value of <0.05 statistically significant. Analyses were conducted in R (R Studio), version 1.1.38.

*Association Between Prevalent TB Infection and Proxies for Household TB Transmission*: To evaluate the hypothesis that proxies of household transmission would have a stronger association with TB infection in younger children (age 5–11 years) than adolescents (12–19 years), we performed age-stratified analyses for proxies of household transmission. The following predictors were defined as proxies for household transmission in children and young adults: household TB contact, mother with a positive TST, and >1 other child in the household with a positive TST.

*Population Attributable Fraction (PAF) of Prevalent TB Infection Due to a Known Household TB Contact*: To further assess age-related variations in the role of household TB exposure and prevalent TB infection in youth we estimated the PAF of prevalent TB infection due to a household TB contact among all youth (5–19 years) and within age strata: 5–11 years and 12–19 years. The PAF is defined as the difference between the observed prevalence of TB infection and expected prevalence of TB infection in the absence of household contacts, divided by the observed prevalence.[13] All estimates used inverse-probability weighting to account for the sampling scheme and incomplete TST placement. The estimates of the expected prevalence of TB infection if there were no household contacts were adjusted for age, sex, wealth, and HIV status.

### Ethics

For the SEARCH community health campaigns, all participants 15 years of age or over provided verbal consent with biometric fingerprint confirmation of agreement, parental consent was obtained for children under the age of 15 and waived for mature minors from ages 15–17. For the TB infection survey, we obtained written informed consent from parents and legal guardians for legal for children and mature minors 17 years and younger, and assent was obtained for participants ages 8 to 17 years. Participants 18 years and older provided written consent. This study is in accordance with the Uganda National Council for Science and Technology Guidelines National Guidelines for Research[14] and was approved by the Makerere University School of Medicine Research and Ethics Committee, Uganda National Council for Science and Technology, and the University of California, San Francisco Committee on Human Research.

## Results

### Participant characteristics

**Fig 1:**
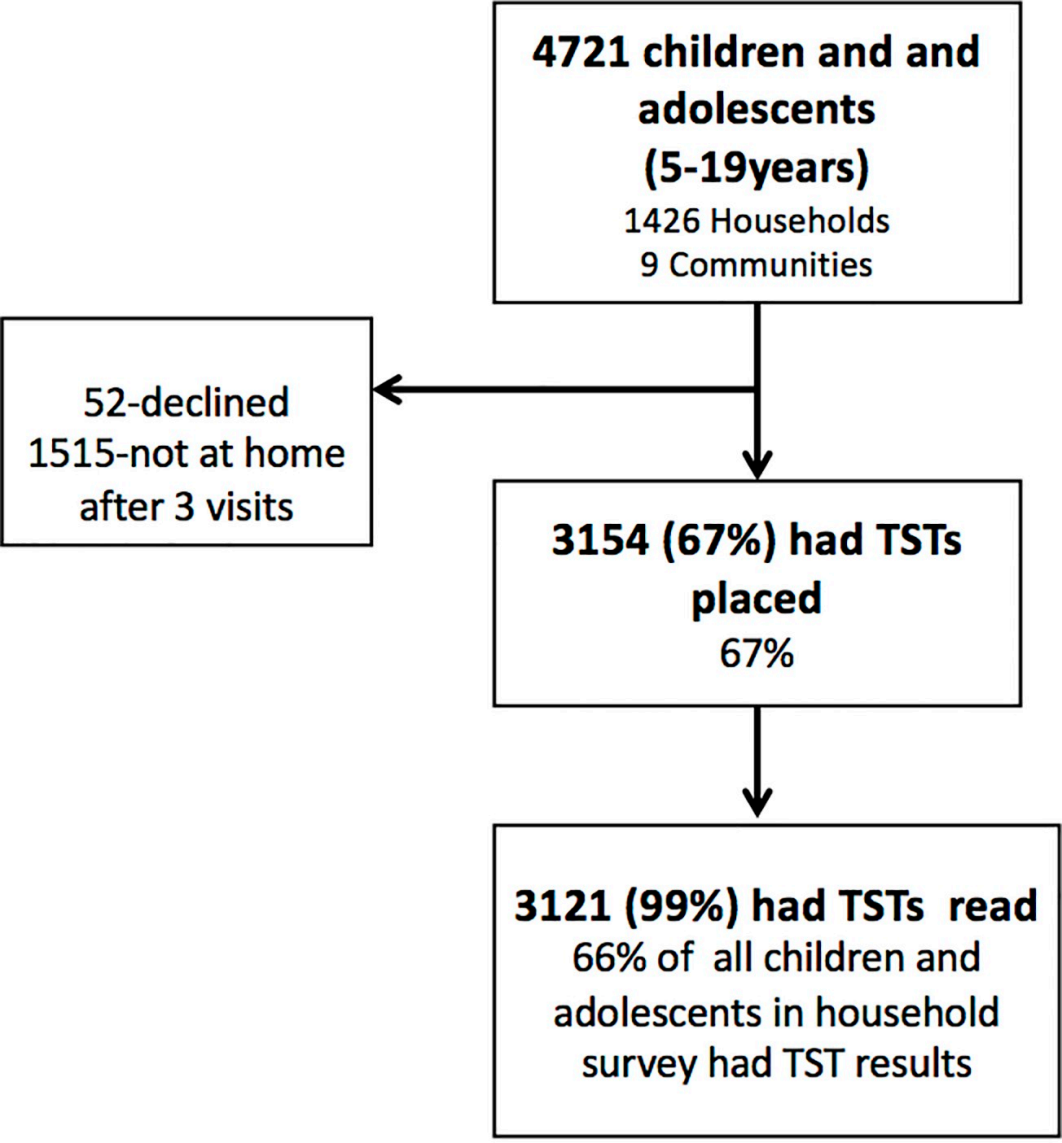
Study flow diagram. TST = tuberculin skin test.

**Fig 2:**
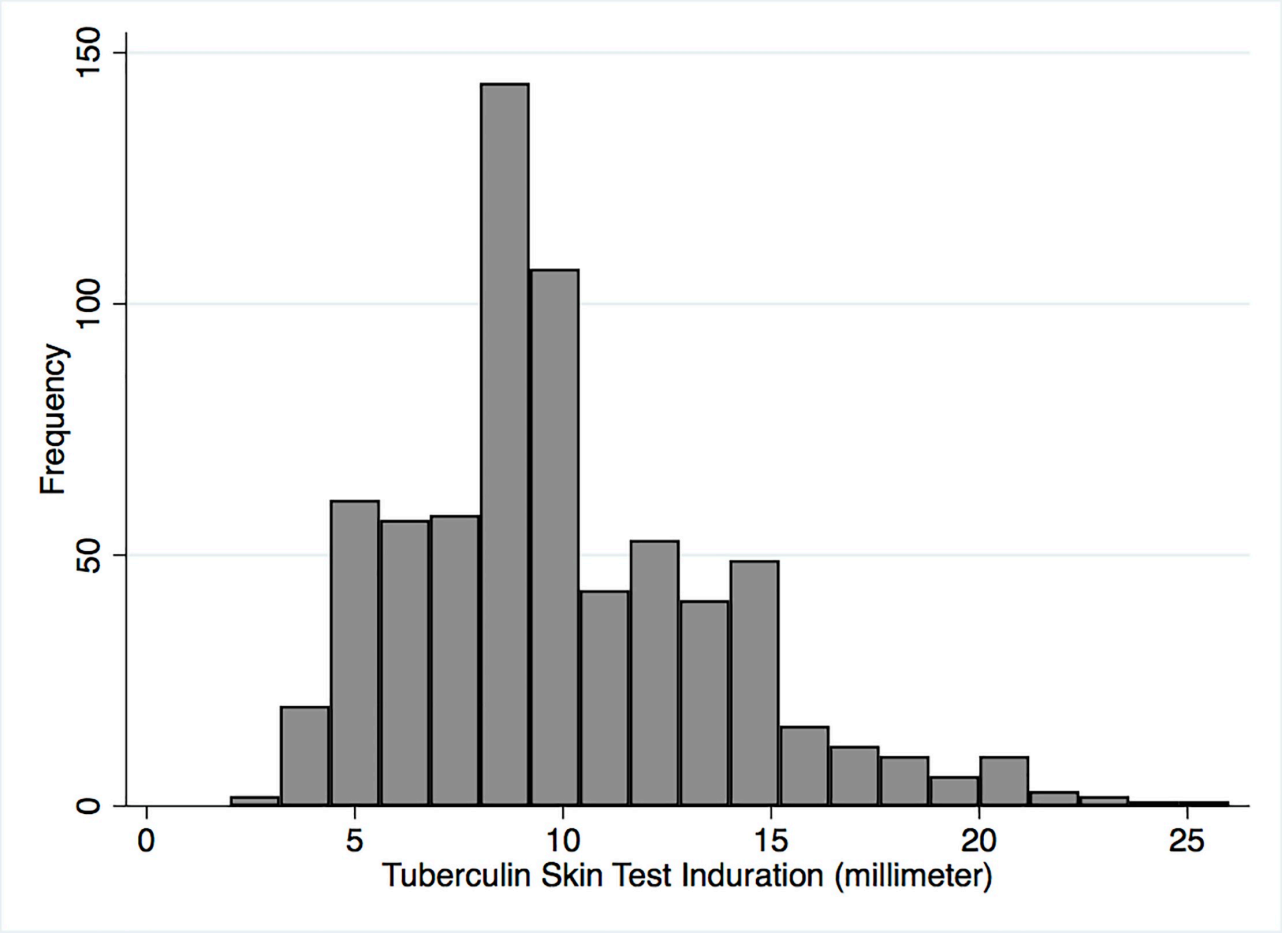
Histogram of non-zero induration from tuberculin skin tests. Among the 3,121 children who had TSTs placed and read, 2425 (77.7%) had an induration of 0 millimeters (mm) and 696 (22.3%) had a non-zero mm induration. The mode of non-zero indurations was 8mm.

TSTs were placed in 3154 of 4721 (67%) youth (ages 5–19 years) living in the 1426 households participating in the nested study. Of the 3154 TST’s placed, 3121 (99%) were read within 72 hours (Fig 1). Demographics and bivariate associations between multi-level predictors are presented in Table 1. The median age of participants was 11 (IQR 7–14), 48.4% were female, 1.2% were living with HIV, 94.8% had evidence of BCG vaccination (scar or record of vaccination), 3.5% reported a household TB contact, and 0.1% reported a prior diagnosis of TB disease. School attendance among youth was 77.4%, the majority of youth attend a public school (64.1%), and 8.0% live outside of the community to attend school. The frequency of non-zero indurations is shown in Fig 2 and the mode was 8mm.

**Table 1:** Characteristics of youth 5–19 years of age participating in the TB Infection Household Survey in 9 rural communities in Uganda.

	Total Population N = 3121	% TST Positive N = 355	
	n(%)	n (%)	p-value
***Individual-Level Variables***			
Median Age (IQR)	11 (7–14)	11 (8–15)	<0.01
5–9	1306 (42)	104 (8)▵	
10–14	1092 (35)	109 (10)▵	
15–19	723 (23)	142 (20)▵	<0.01
Gender			
Male	1612 (52)	187 (12)	
Female	1509 (49)	168 (11)	0.68
Lowest wealth tertile			
No	2504 (81)	263 (11)	
Yes	571 (19)	90 (16)	<0.01
HIV status			
negative	2785 (89)	322 (12)	
positive	35 (1)	2 (6)	
unknown/not tested	301 (10)	31 (10)	0.55
Mother with HIV (possible HIV-exposed uninfected child)*			
No	1927 (80)	105 (8)	
Yes	471 (27)	46 (9)	0.42
BCG vaccinated (scar or record)			
No	163 (5)	18 (11)	
Yes	2598 (95)	337 (11)	0.89
History of TB Disease			
No	3119 (99.9)	353 (11.3)	
Yes	2 (0.1)	2 (100)	<0.01
***Household-Level Variables***			
Characteristics of Household Members	*** ***	** **	** **
Household member living with HIV			
No	1666 (53)	188 (11)	
Yes	1455 (47)	167 (12)	0.57
≥1 household member lives outside of community for 6 months or more			
No	2630 (88)	218 (8)	
Yes	342 (12)	33 (10)	0.10
≥1 household members drink alcohol			
No	2506 (81)	274 (10)	
Yes	600 (19)	81 (14)	0.08
Proxies of Household TB Transmission			
Household TB contact			
No	3011 (97)	322 (11)	
Yes	110 (4)	33 (30)	<0.01
Mother's TST status **			
negative	729 (42)	42 (6)	
positive	353 (20)	52 (15)	
unknown/mother not tested	661 (38)	56 (9)	<0.01
One or more child in household with a positive TST ***			
0 children	2613 (90)	243 (8)	
1 or more child	265 (9)	107 (40)	<0.01
***School and Community-Level Predictors***			
Currently in School			
No or unknown	707 (23)	81 (11)	
Yes	2414 (77)	274 (11)	0.94
Type of School			
Public	1543 (64)	178 (12)	
Private	863 (36)	95 (11)	0.70
Boarding School or attends school outside community****			
No	2202 (92)	230 (10)	
Yes	192 (8)	38 (20)	<0.01
Works outside of home *****			
No	322 (46)	52 (16)	
Yes	388 (54)	90 (23)	0.01

▵Age-specific proportions of positive tuberculin skin tests (TST) are raw prevalence values and differ from adjusted point estimates.

*Maternal HIV status available for children 5–11 years, N = 1452

**Maternal TST status available for children 5–11 years, N = 1231

***Excludes households with two or fewer children, N = 2746

****Includes youth ≥15 years, N = 720

### Population age-specific prevalence of TB Infection

**Fig 3:**
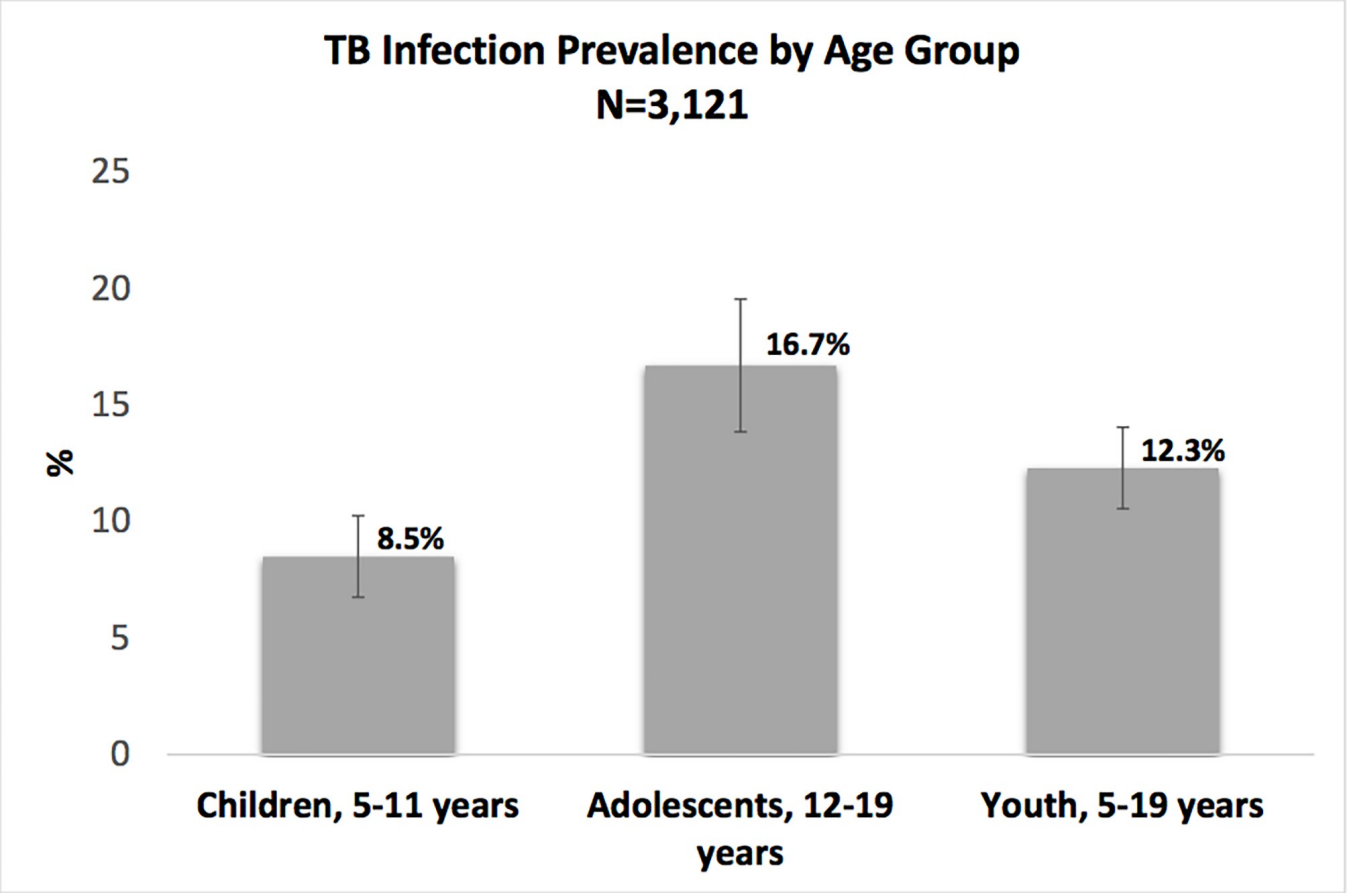
Population-level age-stratified prevalence estimates of TB infection, defined as a positive tuberculin skin test (TST), in duration >10mm or >5mm if living with HIV.

The overall prevalence of TB infection in children and adolescents 5–19 years of age was 12.3% (95% CI: 10.6–14.1), 8.5% (95%CI: 6.9–10.4) in children (5–11 years), and 16.7% (95% CI: 14.0–19.7) in adolescents (12–19 years) (Fig 3).

### Predictors of prevalent TB infection

#### Individual-level predictors

Among all youth, TB infection was positively associated with older age; the adjusted odds of TB were 3.1-fold higher (95% CI:2.2–4.5, p<0.001) for those aged 15–19 years as compared to those aged 5–9 years. Children in the lowest wealth tertile were also at higher risk (aOR 2.0; 95% CI:1.3–2.9, p<0.001). We detected a negative association between HIV seropositivity and TB infection (aOR 0.2; 95% CI: 0.05–0.9, p = 0.04). Prevalent TB infection was not associated with sex, BCG vaccination, or with our proxy for perinatal HIV-exposure (being born to a mother who is currently living with HIV) (Table 2). In both unadjusted and adjusted analyses, similar trends in individual-level predictors were observed across age groups.

**Table 2:** Association between TB infection and individual, household, and community-level predictors in children and adolescents in 9 rural communities in rural Uganda.

	OR(95% CI)	aOR (95%CI)†
***Individual-level factors***		
Age		
5–9	*ref*	*ref*
10–14	1.2 (0.9–1.8)	1.2 (0.9–1.8)
15–19	2.9 (2.0–4.1)****	3.1 (2.2–4.5)****
Gender		
Male	*ref*	*ref*
Female	1.1 (0.8–1.4)	1.1 (0.8–1.4)
Lowest wealth tertile		
Yes	1.9 (1.3–2.8)***	2.0 (1.3–2.9)***
HIV status		
negative	*ref*	ref
positive	0.3 (0.1–1.2)*	0.2 (0.05–0.9)**
unknown/not tested	1.1 (0.5–1.8)	1.2 (0.7–2.0)
BCG vaccination	1.5 (0.7–3.0)	
Mother with HIV (possible HIV-exposed uninfected child)▵	1.0 (0.6–1.6)	
***Household-Level Factors***		
Proxies of Household TB Transmission		
Household TB contact	3.4 (1.3–8.9)**	3.2 (1.3–7.6)****
Mother's TST status ▵		
negative	*ref*	*ref*
positive	2.3 (1.3–4.0)***	2.2 (1.2–3.9)***
unknown/mother not tested	1.6 (0.9–2.3)*	1.5 (0.9–2.6)
Another child (age<15 years) in household with positive TST Ω		
0 children	*ref*	*ref*
1 or more child	7.4 (5.2–10.6)****	6.5 (4.6–9.2)****
Characteristics of Household Members		
Household member with HIV	0.9 (0.7–1.3)	
≥1 household member lives outside of community for 6 months or more	1.2 (0.8–1.8)	
> = 1 household members drink alcohol	1.4 (1.0–1.9)**	1.4 (1.0–1.9)*
***School and Community-Level Factors***		
Boarding School or attends school outside community	2.4 (1.4–3.6)****	1.7 (1.0–2.9)**
Occupation outside of home (age ≥15 years)	1.6 (1.0–2.5)**	1.2 (0.7–2.1)

OR = Odds Ratio; aOR = adjusted Odds Ratio; CI = Confidence Interval; ref = reference group; BCG = Bacilli Calmette Guérin; TST = tuberculin skin test

^†^adjusted by age, gender, wealth, HIV status, and household TB contact

▵Only available for children 5–11

* p-value: ≤0.1

**p-value: ≤0.05

***p-value: <0.01

****p-value: <0.001

#### Household-level predictors and proxies of household transmission

Overall 110 (3.5%) youth (ages 5–19 years) had a known household TB contact. The proportion of youth with TB infection who had a reported a household TB contact was 9% among youth ages 5–19 years, 13% among children ages 5–11 years, and 7% among adolescents ages 12–19 years. Overall, children with a household contact with TB disease had a higher odds of prevalent TB infection compared to those who did not have a contact with TB disease (aOR 3.2; 95% CI: 1.3–7.6, p<0.01) (Table 2). As hypothesized, this relationship was modified by age-group: aOR 5.5, 95% CI: 1.8–16.0, p<0.01 in children and aOR: 2.3; 95% CI:0.8–7.0, p = 0.13 in adolescents.

Prevalent TB infection was also associated with other proxies of household transmission. Prevalent TB infection was positively associated with having another child (5–15 years of age) in the house with TB infection (aOR 6.5, 95%CI: 4.6–9.2, p<0.001), and this association was also modified by age-group (in children: aOR 9.5, 95%CI: 5.9–15.6 and in adolescents: aOR 3.4, 95%CI: 2.0–5.6, <0.001). Prevalent TB infection was also associated with having a mother with a positive TST (aOR 2.2, 95% CI: 1.2–3.9, p<0.001) among children twelve years of age and younger (Table 3).

**Table 3:** Association between prevalent TB infection in youth and proxies for household TB transmission, stratified by age group.

	Children, 5–11 years		Adolescents, 12–19 years		All Youth, 5–19 years		Test for homogeneity across strata
	5–11 years		12–19 years		5–19 years		p-value
Variable	OR (95% CI)	aOR (95% CI) ^⍴^	OR (95% CI)	aOR (95% CI) ^⍴^	OR (95% CI)	aOR (95% CI) ^⍴^	
**Household TB contact**							
No	*ref*	*ref*	*ref*	*ref*	*ref*	*ref*	
Yes	5.5 (1.7–16.9)****	5.5 (1.8–16.0) ***	1.3 (0.5–3.2)	2.3(0.8–7.0)	3.4 (1.3–8.9) *	3.2 (1.3–7.6) ****	0.048
**Mother's TST status**		** **					
negative	*ref*	*ref*	n/a	n/a	n/a	n/a	
positive	2.3 (1.3–4.0) **	2.2 (1.2–3.9) **	n/a	n/a	n/a	n/a	
unknown	1.6 (0.9–2.3) *	1.5 (0.9–2.6)	n/a	n/a	n/a	n/a	
**Another child in household with positive TST Ω**						
No	*ref*	*ref*	*ref*	*ref*	*ref*	*ref*	
Yes	10.2 (6.0–17.1) ****	9.6 (5.9–15.6) ****	4.2 (2.5–6.9) ****	3.4 (2.0–5.6) ****	7.4 (5.2–10.6) ****	6.5 (4.6–9.2) ****	0.001

OR = Odds Ratio, aOR = adjusted odds ratio, CI = Confidence Intervals

^⍴^ adjusted by age (by year), sex, HIV status, TB contact, and lowest wealth tertile

* p-value: ≤0.1

**p-value: ≤0.05

***p-value: <0.01

****p-value: <0.001

n/a = not available, as data only for children <12 years

Characteristics of household members (HIV status, high mobility, and alcohol use) were not associated with prevalent TB infection in adjusted models across age groups (Table 2), though the prevalence of active TB disease (up to a year prior to TST placement) and reported household TB contact was higher in households with a household member living with HIV compared to those without: 2.9% vs. 0.4% (p = <0.01) and 4.9% vs. 3.1% (p<0.001) respectively. There was a positive trend in the association between prevalent TB infection and living with a household member who drinks alcohol (aOR: 1.3, 95% CI: 1.0–1.9, p = 0.07) (Table 2), and this trend was consistent across age-groups.

#### Community and school-level predictors

Attending school and whether the school was private or public was not associated with prevalent TB infection, p = 0.94 and p = 0.70 respectively (Table 1). Among youth, attending school outside of the community and this was positively associated with prevalent TB infection (aOR 1.7, 95% CI 1.0–2.9, p = 0.03). Among youth, 15–19 years of age, having a job outside of the farm or household was associated with an increased odds of TB in univariate models, but this was not significant in adjusted models (aOR: 1.2 95% CI: 0.7–2.1, p = 0.41) (Table 2). In both unadjusted and adjusted analyses, similar trends in community-level and school-level predictors of prevalent TB infection were observed for across age groups.

#### Population attributable fraction of TB infection attributable to a known household TB contact

The population attributable fraction of prevalent TB infection due to household TB exposure among youth ages 5–19 years was 3.8% and varied by age group: 8.1% in children ages 5–11 years, 3.9% in adolescents 12–19 years. (S1 Table).

## Discussion

Data on the age-specific prevalence and risk factors for TB infection in children and adolescents in rural communities are sparse and needed to inform TB control efforts. In this cross-sectional, community-representative study of over 3000 children and adolescents living in rural Uganda we found a high prevalence of TB infection, with 9% of children and 17% of adolescents already infected with TB. We found a strong positive association between prevalent TB infection and proxies of household TB transmission, however, the strength of this association decreased among adolescents. Our findings, along with prior studies from urban areas of South Africa [6,15], suggest that the importance of household TB transmission on TB acquisition declines as children age into adolescence. Additionally, on a community-level, only 8% of TB-infections in children and 4% of TB infections in adolescents were attributable to known household TB contacts. These findings are consistent with a meta-analysis of children in high-TB burden settings, which estimated that on a population-level household transmission accounted for <20% of TB infections.[5] Despite the importance of transmission outside of the home, data on community-based risk factors for TB infection in youth are sparse. Our findings suggests that mobility among youth, specifically to attend boarding schools and schools outside the community, may be an novel risk factor for acquiring TB infection outside of the home.

This is one of the first studies to assess the age-specific relationship between proxies of household TB exposure and TB infection in youth living in rural communities of East Africa, where social-mixing patterns and TB transmission dynamics may differ from those in urban centers. Children with a household TB contact had a 5.5-fold adjusted odds of having prevalent TB infection compared to those who did not, however, in adolescents this association was weaker association and did not meet statistical significance. Similarly, age also modified the association between prevalent TB infection in youth and a second proxy for TB transmission, the presence of another child in the household with a positive TST. These findings are consistent with a modeling studies from South Africa that attribute the age-related shift from household to community-based TB transmission to an increase in the number of indoor hours spent in community-based aggregate settings and to changes in social-mixing patterns[7,15]. In South Africa, schools and transport are thought to be the predominant sites of TB acquisition in children,[15,16] however, additional sociospatial mixing and ventilation studies are needed to pinpoint where in rural communities youth are acquiring TB.

Mobile youth may be at an increased risk of TB infection. We found that children traveling outside of the community for school had a 2-fold higher odds of prevalent TB than those who attended rural schools in their communities. Leaving the community to attend school, usually to attend boarding schools, is common in rural areas of Uganda, where approximately 50% of school-going children attend private schools and boarding schools[17]. Environmental conditions of these schools may be rife for TB transmission, as there may be worse ventilation and more crowding in boarding schools compared to rural day schools. Additionally, youth who leave the community to attend school may have a higher risk of TB because of mobility-related risk factors, such as (1) increased risk of TB exposure during transport to outside communities or within urban centers [16] and (2) expansion of a youth’s social network into an area of higher TB prevalence. Further work is needed to assess patterns of mobility in children and their relationship to TB acquisition, as mobile youth may benefit from more intensive TB screening.

HIV fuels a significant portion of the TB epidemic in sub-Saharan Africa [18,19]; however, less is known about the extent to which adult HIV infection drives TB acquisition in children. We did not see an association between prevalent TB infection in youth and living in a household with an adult with HIV, even though TB disease was more common in households with PLWH than those without. This lack of association may be because in households with PLWH the risk conferred by a higher prevalence of TB is offset by lower TB infectiousness among PLWH [20].

In early childhood, HIV-exposure has been associated with an increased odds of TB infection [21], but this relationship has not been examined in older children. We did not find an association between prevalent TB infection and our proxy for perinatal HIV-exposure (having a mother with HIV). One possible explanation for this finding is that the immunologic differences between children exposed to HIV in the perinatal period compared to those who are not exposed diminish as children age [22]. These differences likely diminish as children age. Though maternal HIV-exposure was not associated with TB, children whose mothers had a positive TST had a higher odds of TB infection. This finding highlights the importance of family-based TB screening and integration of TB prevention and screening into maternal and child health services.

Our data adds to the literature by providing community-representative estimates of the burden of TB infection in adolescents in East Africa, a high priority group for which few estimates are published. Our estimate of prevalent TB infection in adolescents of 17% is similar to the one other TST survey in rural areas in Uganda[23], but lower than those from South Africa and urban areas in Uganda. Though not directly comparable due to differences in sampling and age-strata, TST positivity was 16% in a convenience sample of over 5000 adolescents (12–18 years) in rural Uganda and 55% in over 5000 adolescents (12-18-year-old) in South Africa. The force of TB infection increases rapidly in adolescents[24], and adolescent-specific TB interventions may be needed to combat TB in this high priority group.

To our knowledge this is one of the first studies to estimate a community-representative population attributable fraction (PAF) of prevalent TB infections attributable to household transmission among youth (ages 5–19 years) living rural communities in East Africa. We found that 8% of TB infections among children 5–11 years old and 4% among adolescents (11–19 years) were attributable to a known household TB contact, which is consistent with prior studies that found on a population-level that household transmission only accounted for a small proportion of TB infections in children.[5,25] Our estimates are slightly lower than estimates of 10–30% published in prior studies[5,25], and this is likely because: (1) our sample includes older children and adolescents, whereas prior estimates were from children under the age of five and (2) our estimates likely under-estimates the PAF of household transmission as under-diagnosis of TB disease in rural areas of East Africa is common, and our estimate does not account for household transmission from undiagnosed household contacts.

This study should be interpreted within the limitations of the study design. There is no gold standard of TB infection measurement and TSTs may overestimate the prevalence of TB infection due to the cross-reactivity with BCG vaccination and exposure to environmental non-tuberculous mycobacteria. However, we suspect minimal cross-reactivity with non-tuberculous mycobacteria (NTM) as our distribution of non-zero TSTs was symmetric, suggesting little sensitization to NTM. Though BCG can cause false positive TSTs, we used an induration cut off of 10mm to minimize this effect and the effect of BCG on TST size is minimal after the age of 5[26]. Household surveys on TB infection can underestimate infections as persons who are at high risk for TB infection may not be at home (ie. mobile youth, men, PLWA). We were able to account for sampling bias and to provide population-level estimates by using inverse probability treatment weighting, as our study was nested within the SEARCH study, which had detailed demographic data about the persons in our sample who did not have a TST placed. There are some limitations to our age-stratified analyses. Older children and their parents may be less likely to recall a TB exposure that occurred years ago, as opposed to school-age children who may have had a more recent exposure. Finally, this is a cross-sectional study and our analysis of predictors of TB infection cannot assess causality.

In conclusion, this study reported a high prevalence of TB infection in youth, especially in adolescents. In adolescents TB infection was not significantly associated with a known household contact and overall on a population-level household TB contacts accounted for a small proportion of TB infections. These findings highlights the importance of community-based transmission as an important site for TB acquisition in this vulnerable high-priority risk group. While expectedly TB infection was associated with individual-level risk factors described previously (age, poverty, and wealth), our data suggest that mobile youth who leave the community for school are at higher risk of TB and so are a potential novel risk group for TB acquisition. Overall, these data underscore the need for community-based interventions that account for age-related variations in social and spatial networks to prevent TB among rural children living in East Africa.

## Supporting information

S1 Table Population Attributable Fraction (PAF) of prevalent TB Infection due to household TB exposure by age group. PAF = (E-O)/E. O = Observed prevalence of TB infection E = Expected prevalence of TB infection in the absence of known household TB contacts. All prevalence estimates used inverse-probability to account for sampling scheme and incomplete TST placement and the estimates of the expected prevalence if there were no household contacts were adjusted for age, sex, wealth tertile, and HIV status.
(DOCX)
