# Supplementary material for: The age-specific burden and household and school-based predictors of child and adolescent tuberculosis infection in rural Uganda
Source: PLoS One. 2020 Jan 29;15(1):e0228102. doi: 10.1371/journal.pone.0228102 (PMC6988961; doi:10.1371/journal.pone.0228102)
Supplement: S1 Table — PAF = (E-O)/E. O = Observed prevalence of TB infection E = Expected prevalence of TB infection in the absence of known household TB contacts. All prevalence estimates used inverse-probability to account for sampling scheme and incomplete TST placement and the estimates of the expected prevalence if there were no household contacts were adjusted for age, sex, wealth tertile, and HIV status. (DOCX) [file pone.0228102.s001.docx]

| Age Group | **O=** Observed prevalence of TB infection | **E=** Expected prevalence of TB infection if no known household contacts | Population Attributable Fraction= (E-O)/E |
| --- | --- | --- | --- |
| 5-11 years | 8.5% | 7.8% | 3.8% |
| 12-19 years | 16.7% | 11.8% | 8.1% |
| 5-19 years | 12.3% | 16.1% | 3.9% |
